# Bioinspired Nanostructured Superwetting Thin-Films in a Self-supported form Enabled “Miniature Umbrella” for Weather Monitoring and Water Rescue

**DOI:** 10.1007/s40820-021-00775-4

**Published:** 2021-12-13

**Authors:** Shan Li, Peng Xiao, Wei Zhou, Yun Liang, Shiao-Wei Kuo, Tao Chen

**Affiliations:** 1 Key Laboratory of Marine Materials and Related Technologies, Zhejiang Key Laboratory of Marine Materials and Protective Technologies, Ningbo Institute of Materials Technology and Engineering, Chinese Academy of Sciences, Ningbo, 315201 People’s Republic of China; 2 School of Chemical Sciences, University of Chinese Academy of Sciences, 19A Yuquan Road, Beijing, 100049 People’s Republic of China; 3 Department of Material and Optoelectronic Science, National Sun Yat-Sen University, Kaohsiung, 804 Taiwan, People’s Republic of China

**Keywords:** Self-supported, Superhydrophobic thin films, Multifunctional sensing, Water rescue

## Abstract

- An elastic, superhydrophobic and conductive thin film inspired by the natural self-supported superhydrophobic butterfly wings enabled by a controllable composite of assembled carbon nanotube and elastomer is fabricated.
- Through the adjustment of hydrophobic elastomeric coating, the surface wettability can be effectively controlled and still maintain superhydrophobic characteristics under the applied strain of 60%.
- The achieved film can function as a self-supported smart umbrella to sensitively monitor the day weather and perform water rescue

**Abstract:**

Two-dimensional (2D) soft materials, especially in their self-supported forms, demonstrate attractive properties to realize biomimetic morphing and ultrasensitive sensing. Although extensive efforts on design of self-supported functional membranes and integrated systems have been devoted, there still remains an unexplored regime of the combination of mechanical, electrical and surface wetting properties for specific functions. Here, we report a self-supported film featured with elastic, thin, conductive and superhydrophobic characteristics. Through a well-defined surface modification strategy, the surface wettability and mechanical sensing can be effectively balanced. The resulted film can function as a smart umbrella to achieve real-time simulated raining with diverse frequencies and intensity. In addition, the integrated umbrella can even response sensitively to the sunlight and demonstrate a positively correlation of current signals with the intensity of sun illumination. Moreover, the superhydrophobic umbrella can be further employed to realize water rescue, which can take the underwater object onto water surface, load and rapidly transport the considerable weight. More importantly, the whole process of loaded objects and water flow velocity can be precisely detected. The self-supported smart umbrella can effectively monitor the weather and realize a smart water rescue, demonstrating significant potentials in multifunctional sensing and directional actuation in the presence of water.
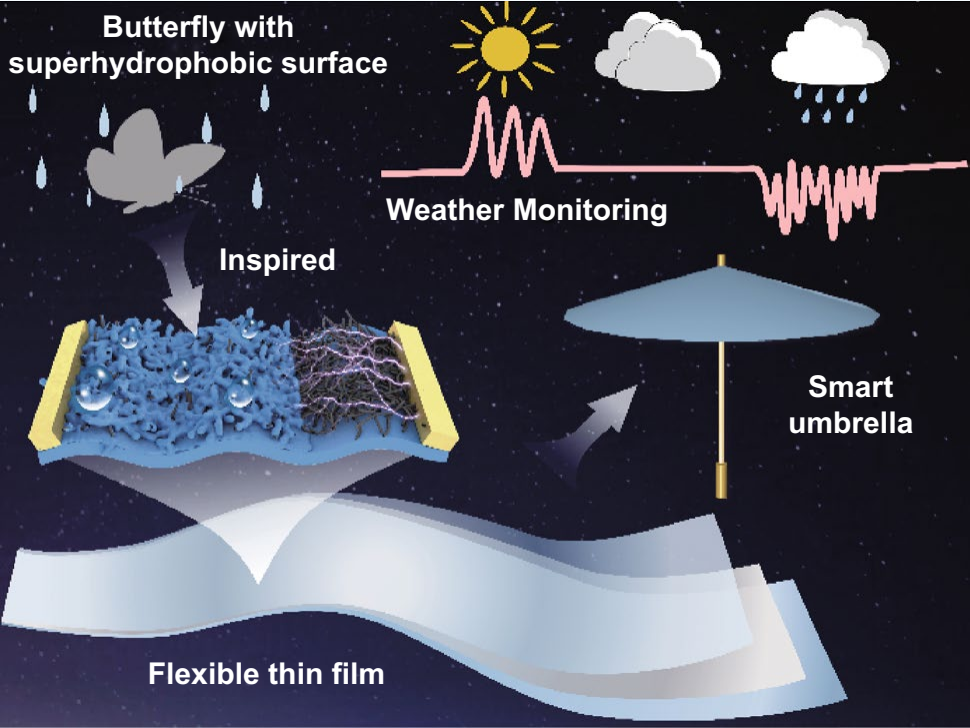

**Supplementary Information:**

The online version contains supplementary material available at 10.1007/s40820-021-00775-4.

## Introduction

Soft materials, especially the two-dimensional (2D) ones, have drawn extensive attentions due to their highly flexible, morphable and self-adaptable features, demonstrating broad potential applications in soft actuation [1–5], wearable mechanical sensing [6–13] and flexible energy storage [14–17]. In particular, 2D membranes in their self-supported forms are considered to display some exceptional properties that differentiate from the supported ones [18–21]. Nature has given us some interesting cases of self-supported membranes with specific functions. A typical example of ultrathin eardrum that can be effectively driven to experience regular vibration for the precise transmission of the external acoustical signals [22, 23]. In addition, some male frogs have thin, elastic and self-supported membranes as sound sac, which can be pneumatically inflated to control their singing behaviours [10].

Based on these impressive phenomena, some great efforts have been dedicated to design self-supported membrane systems to realize biomimetic functions such as controllable camouflage elastic skins [24, 25], shape-morphing systems [26–28], and magnetic technology-based tactile sensors [29]. For example, self-supported stretchable surfaces with patterned inextensible textile mesh on elastomeric membranes were developed to achieve pneumatic actuation from 2D to hierarchical three-dimensional (3D) camouflage [30, 31]. For the sensory system, a self-supported membrane of magnetic particles and elastomeric matrix composite was introduced into a tactile sensor with well-designed air gap for smart prosthetics [32]. Therefore, the self-supported systems have provided a novel and alternative pathway for biomimetic actuation and soft electronics. However, when exposed to water environment, it is difficult to sensitively and stably capture the tiny/large mechanical stimuli and give a real-time response. Moreover, current researches mostly focus on the construction of superhydrophobic mechanical sensors targeted on supported membranes or bulk aerogels. For the self-supported 2D thin-membranes, their potential is severely limited by a lack of effective integration of diverse functional components. Achieving integrated membranes with ultrathin thickness, conductivity, stretchability and superwettability into one system has been a challenge, which is expected to adapt to changeable water-related environment [33, 34].

Water/air interface is an important functionalization platform for well-controlled self-assembly and two-dimensionally confined asymmetric physical/chemical reactions. Compared with the conventional method, such as the spin coating, the membranes prepared by the interface strategy are ultrathin, and easily transferred to various substrates for diverse applications. In addition, the interface fabrication strategy with simple and controllable features enables large-scale and efficient production. Therefore, the water/air interface provides an ideal platform for the preparation of functional two-dimensional thin membranes.

In nature, the example of some butterflies with superhydrophobic self-supported wings has enlightened people to study and imitate the interesting phenomenon [35, 36]. In rainy weather, the self-supported superhydrophobic wings of butterfly can effectively repel water droplets and maintain ordered flapping, resulting in a stable light flight even under rainy environment [37]. Similarly, for the mechanical sensory systems that are sensitive to water, the introduction of superhydrophobic and ultrathin features is expected to endure water environment for sensitive/stable sensing function and beyond. Here, inspired by the natural self-supported superhydrophobic butterfly wings [38, 39], we develop an elastic, superhydrophobic and conductive thin film enabled by a controllable composite of assembled carbon nanotube and elastomer for a smart umbrella. Through the adjustment of hydrophobic elastomeric coating, PDMS/CNTs/PDMS composite membrane (PCPM) with asymmetric three-layer structure was prepared. The surface wettability can be effectively controlled and still maintain superhydrophobic characteristics under the applied strain of 60%. The achieved film can function as a self-supported smart umbrella to sensitively capture the simulated rain droplets with a series of frequencies and intensity. Moreover, the self-supported smart umbrella can even response to the solar intensity for weather monitoring. More interestingly, owing the superhydrophobic feature, the umbrella can act as water rescue device to take the objects out of water and enable a fast motion across the water surface. In addition, it can even realize a sensitive monitoring of the water flow velocity and the change of loaded objects, demonstrating significant potentials in water rescue.

## Experimental Section

### Materials

The raw carbon nanotubes (CNTs) (length, about 10–30 μm; diameter, about 10–30 nm; –COOH %, about 2 wt%) with a purity of over 90% were acquired from Chengdu Organic Chemistry Co., Ltd, and were rinsed thoroughly with anhydrous ethanol and dried in a stream of nitrogen before use. PDMS film was fabricated from Sylgard 184 (the ratio of base-to-curing was 10:1). Prior to film formation, the PDMS and corresponding cross-linker were diluted using n-heptane with a weight ratio of 4 wt%. General chemicals in chemical reagent grade were used as received from Sinopharm Chemical Reagent. The PET (thick, about 0.5 mm) was rinsed with deionized water and ethanol, then dried with a nonwoven cloth, and used as a transfer substrate.

### Preparation of CNT Film

First, 1 g of the CNTs-COOH was dispersed in 500 mL anhydrous ethanol, followed by ultra-sonification for all night. Then, 30 mL of ethanol-assisted carbon materials suspensions were spread onto the water surface by a spray-coating method, resulting in a uniform preassembled film formed at the air/water interface. To achieve a homogeneous film, the location of the sprayer should be orderly changed during the spray-coating process. Subsequently, a microporous sponge was used to put on one side of the water/air interface to quickly siphon water from the system, followed by a prominent decrease in the preassembled CNT film area. Notably, the homogeneous preassembled CNT film was closely packed towards the opposite direction of the siphon direction. When the move behaviour of the film stopped and further siphon cannot drive the film, the resulting film is ultimately formed.

### Preparation of PDMS Film

60 mL of the mixture of 4 wt% PDMS in n-heptane was sprayed onto the air/water interface to achieve a homogenous layer, followed by a typical PDMS curing process (at 80 °C for 3 h) to form a uniform PDMS film.

### Fabrication of PCPM

First, the PDMS film prepared above was transferred to the PET substrate in order to facilitate the subsequent transfer of CNTs film. Then, CNTs with different layers (one, two and three) were transferred to PDMS film to achieve superhydrophobicity of hybrid film. To protect the surface of CNTs without affecting their rough hydrophobic structure, the CNTs side were uniformly sprayed with an ultrathin layer of PDMS (mass fraction, 2 wt%; volume, about 1 mL). Subsequently, the film was cured at 60 °C for 2 h, and a superhydrophobic, self-adhering PCPM was formed.

### Fabrication of PCPM Smart Umbrella

Oilpaper umbrellas with a diameter of 10 cm were cut with commercial scissors to get the umbrella frame. To make the frame strong robust, a commercial 502 glue was applied to the joint of the frame. Then, the PDMS and CNTs film were transferred to the umbrella frame at the air/water interface to obtain the self-supporting hybrid film. Noted that after each layer of CNTs film was transferred, it needed to be placed in the air for 1 h first to make water naturally evaporate from the top to the edge, so that CNTs could be bonded closely. Then, it was transferred to a vacuum dryer and dried at 60 °C for 2 h. If the CNTs are dried in a vacuum oven at the beginning, the water between the CNTs layers evaporates too fast to release water vapour from the top or bottom, which will cause the CNTs film to burst in the middle, thus greatly reducing its electrical conductivity. Finally, an ultrathin PDMS coating was sprayed onto hybrid membranes to protect the CNTs, and a superhydrophobic, self-supporting smart umbrella was prepared.

### Characterization and Measurements

Scanning electron microscopy (SEM) was performed to observe the micromorphology of PCPM with a Hitachi S4800 cold field emission SEM at an accelerating voltage of 4 kV. Energy-dispersive X-ray spectroscopy (EDS) was used to obtain cross section element information of PCPM at an acceleration voltage of 8 kV. The wettability of the membrane was performed on the contact angle measuring instrument (OCA20, America) at room temperature. Five different positions were measured for each sample. When characterization of the sensing performance of the PCPM, an Instron 5567 universal testing machine was used to stretch PCPM rectangular specimens (20 × 5 mm^2^) with one end fixed and the other end linearly elongated at a constant speed. Resistance measurements were carried out by connecting the two ends of the PCPM to an Electrochemical Workstation (CH Instruments, CHI660E.Chenhua Co., Shanghai, China), with conductive copper wires to record the real-time current (*I*) flowing through the film under a constant voltage (*U*_0_) of 1 V, while the real-time resistance (*R*) was calculated by the equation *R* = *U*_0_/*I*. The IR images of the PCPM smart umbrella were measured by an infrared thermal imager (TG165, FLIR, US). AFM measurements were conducted using Dimension ICON SPM (Bruker, USA) in a PeakForce tapping mode.

## Results and Discussion

### Fabrication and Application of Bioinspired Self-supported PCPM Thin-films

**Fig. 1:**
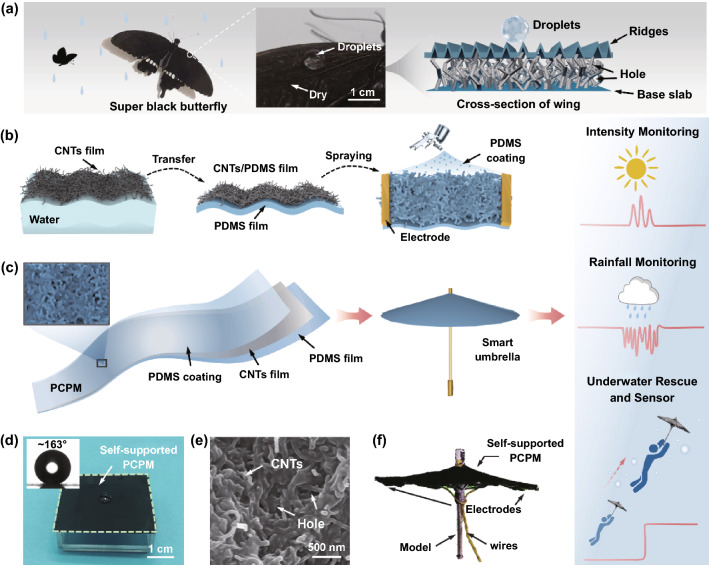
**a** Schematic of super black butterfly wing structure and superhydrophobic principle in rain. **b** Diagram of the preparation of bionic structured PCPM. **c** Schematic of PCPM’s structure and its application in smart weather monitoring and sensing. **d** Photograph of water droplets on the self-supporting, thin PCPM. Inset: Water contact angle (WCA) of the PCPM. **e** SEM image of PCPM coated with 2 wt% PDMS. PCPM is composed of numerous holes and nanoscale CNTs bridges. **f** The structure composition of PCPM smart umbrella

Nature has given us interesting cases of self-supported functional surfaces. An example of super black butterfly wing that shows flexible, self-supported and superhydrophobic properties enables a continuous flying behaviour in the rain. As shown in Fig. 1a, the surface structure of super black butterfly wings consists of three parts: ridges, hole and base slab. Ridges have a hierarchical structure consisting of submicron pore arrays and nanosized ridges [40]. This hierarchical structure combined with the holes can effectively prevent water droplets from infiltrating the wings, thus enabling the water to slip away from the surface quickly. The wings with superhydrophobic feature have inspired us to introduce surface superwettability into the self-supported film system. Through an interfacial self-assembly of carbon nanotubes (CNTs), interfacial curing of elastic elastomer and final spray-coating of hydrophobic elastomer, an elastic, thin, conductive and superhydrophobic film can be successfully fabricated (Fig. 1b). In detail, the uniform CNTs film with entanglement conductive network was formed at the water/air interface through the interface self-assembly based on Marangoni effect and the capillary force driving compression strategy [41]. Compared with unmodified carbon nanotubes, CNTs-COOH has better dispersion in ethanol solution (Fig. S2). Therefore, the CNTs-COOH was selected in our work. Subsequently, the PDMS solution in heptane was spread on water surface for homogenous thin film. Through the adjustment of layers of CNTs films transferred onto thin PDMS film, CNTs/PDMS composite film with different hydrophobic properties, roughness and electrical properties can be obtained. Figure S1 shows the SEM image of the composite film with one, two, and three layers of CNTs transferred onto the PDMS surface. It is clearly observed that with the increase in transferred layers of CNTs film, the achieved surface becomes more and more rough. In addition, the corresponding surface hydrophobility also demonstrates a remarkable improvement (Fig. S3). Moreover, when increasing the CNTs layers, the resulted resistance presents a gradual decrease tendency. As a result, given the balance of conductivity, wettability and fabrication feasibility, three layers of CNTs film were chosen in our system.

More importantly, to acquire a more stable superhydrophobic characteristics, hydrophobic PDMS solution was uniformly coated on CNTs film surface via a facile spray-coating strategy (Fig. 1b). After typical curing procedure, a superhydrophobic composite film composed of PDMS/CNTs/PDMS (PCPM) was finally achieved. Microscopically, the appropriate introduction of PDMS coating cannot prominently affect the surface roughness. On the contrary, the PDMS coating is just like a “armour” to protect the surface from possible breakage (Fig. S4). As a result, the achieved PCPM in a self-supported form can further function as a smart umbrella to realize multifunctional applications, including weather monitoring and water rescue (Fig. 1c). As shown in Fig. 1d, a self-supported film with superhydrophobic was achieved, enabling a water contact angle (WCA) of ~ 163°. Owing to the refined modification of PDMS coating, a rough microstructure can be effectively maintained via a refined control of the surface morphology (Fig. 1e). As a result, a self-supported PCPM enabled smart umbrella is carefully designed in Fig. 1f, which represented good adaptive property on the umbrella skeleton.

### Superhydrophobic Properties and Stability of PCPM

**Fig. 2:**
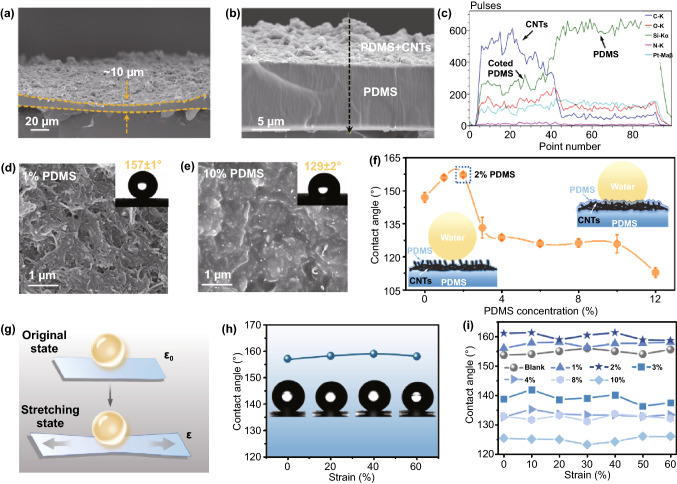
**a** Cross-sectional SEM images of the PCPM. **b**–**c** Energy dispersive spectrometer (EDS) section line sweep and element analysis images of the PCPM. **d**–**e** SEM images of the PCPM coated with 1 wt% and 10 wt% PDMS and their corresponding WCA images. **f** The water contact angles of PCPM coated with PDMS at different mass concentrations. Inset: hydrophobic mechanism diagram of PCPM coated with 2 wt% PDMS (left) and 10 wt% PDMS (right). **g** Schematic diagram of the PCPM tensile contact angle test. **h** WCA-tensile strain curve (*ε*: 0, 20%, 40%, 60%) of PCPM coated with 2 wt% PDMS. **i** Relationships of the surface wettability in PCPM samples with a series of PDMS coating concentration under varied strain of 0%-60%

In order to further explore the correlation between superwettability and microstructure, SEM cross-sectional images were conducted in our experiment. As shown in Fig. 2a, a rough surface was formed on one side of the PCPM with the thickness of about 10 μm. In addition, the energy dispersive spectrometer (EDS) was also measured to conduct element analysis of PCPM, resulting in uniform distribution of Si element from PDMS layer on the sample surface (Fig. 2c). The optimized armour-like PDMS thin coating should maintain the rough microstructure of CNTs film and form a continuous polymeric layer for ensuring stable superhydrophobic surfaces. As a result, the water contact angel of the functional CNTs and pure PDMS side presented 163 ± 3 and 119 ± 2°, respectively (Fig. S5). In addition, the morphology of PCPM films coated with 2 wt% PDMS and without PDMS is significantly different (Fig. S6). It was observed that the hydrophobic PDMS at the micron level was coated around the CNTs and that the large holes between CNTs were transformed into small holes with high density. Thus, droplets can be held up by a uniform layer of air and carbon nanotubes without infiltration.

To further investigate the optimized PDMS concentration on the hydrophobic properties of PCPM, different mass concentration of PDMS solution was applied on the CNTs side by spraying and subsequent curing procedure. As shown in Figs. 2d, e and S7, the degree of surface roughness on CNTs side demonstrates a negative relation with PDMS coating concentration. PCPM with 1 wt% PDMS coating was endowed with considerable microstructure, resulting in excellent superhydrophobic performance with the water contact angle of 157 ± 1°. However, when increasing PDMS concentration of 10 wt%, the rough CNTs microstructure was almost completely covered by PDMS, leading to a relatively smooth surface with decreased WCA of 129 ± 2°. Atomic force microscopy (AFM) images further showed that with the increase in PDMS coating concentration, the surface microstructure of PCPM experienced a gradual change trend from rough to smooth state (Fig. S8a). With the increase in PDMS concentration, the calculated surface root mean square roughness (Rq) represented a remarkable decreasing from 136 to 77.5 nm (Fig. S8b). The diagram in Fig. 2f clearly illustrated the tendency between PDMS concentration and resulted water contact angels. It can be concluded that the parabola-like curve further evidences that the optimized PDMS concentration is 2 wt%. And lower and higher concentration can result in incomplete PDMS coating or loss of roughness. In addition, the original roughness of CNTs film was also investigated in our system. It can be observed that with the increase in CNTs film layers, the water contact angel shows a gradual increase trend from ~ 134 to 155° (Fig. S9). Although further PDMS coating can slightly reduce the WCA value, the final PCPM with 3-layer CNTs film can still maintain superhydrophobic feature.

Furthermore, we have also explored the stability of superwettability under the condition of applied strain up to 60%. The corresponding tensile strain (*ε*) is calculated by Eq. (1):

1 $$\varepsilon = \left( {L - L_{0} } \right)/L_{0} \times 100\%$$

where *L* and *L*_0_ are the film length at the tensile state and the relaxed state, respectively. When an uniaxial stretch was applied on PCPM (2 wt% PDMS-coated), as displayed in Fig. 2g, h, the surface wettability of the PCPM remains a stable state with the WCA value of above 155° during the stretching range from 0 to 60%. The results further indicate that the PCPM can be effectively employed to conduct mechanical strain sensing with stable superhydrophobic performance. In addition, we have also conducted systematic study of the surface wettability in PCPM samples with a series of PDMS coating concentration under varied strain of 0–60% (Fig. 2i). It can be found that when a uniaxial strain was applied, PCPM samples with different concentration of PDMS coating could still maintain relatively stable WCA values. As shown in Fig. S10, PCPM films remain tightly entangled under the tensile strain of 10, 20, 40, and 60%, demonstrating that the armour-like PDMS coating can form an elastic protective layer to avoid mechanical strain.

### Strain Sensing Performance of the Strain Sensor

**Fig. 3:**
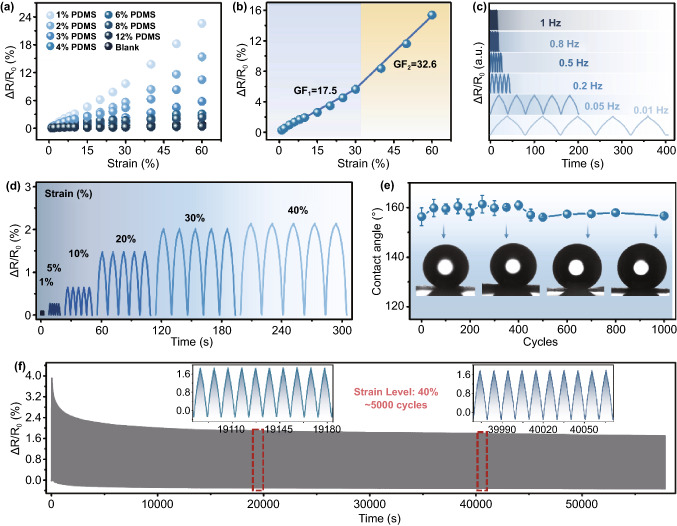
**a** Relative resistance variation of the PCPM coated with different mass concentrations (from 0 to 12 wt%) of PDMS under different tensile strains. **b** The sensing performance of the PCPM (coated with 2 wt% PDMS) with detailed gauge factor. **c**–**d** Relative resistance variation of PCPM under different frequencies (from 0.02 to 1 Hz) between 0 and 40% strain and under different strain (*ε*: 1%, 10%, 20%, 30%, 40%). **e** The water contact angle of the PCPM at different times of cyclic stretching (under *ε* = 40%). **f** The relative resistance changes under cyclic stretching with a frequency of 0.2 Hz over 5000 cycles (under *ε* = 40%)

The sensing performance of PCPM was further characterized, as shown in Fig. 3, a typical plot for the PCPM-based strain sensor was tested at different loading conditions by the normalized electrical resistance (*ΔR*/*R*_0_) change:

2 $$\Delta R/R_{0} = \left( {R - R_{0} } \right)/R_{0}$$

where *R* represents the real-time resistance and *R*_0_ is the initial resistance at the relaxed state (the Janus film strip with lateral dimensions of 20 × 5 mm^2^ (length × width)). In the uniaxial tensile tests accompanied by simultaneous resistance measurements, the sensor with different concentrations of PDMS coating has different relative resistance variation. The corresponding gauge factor (GF) is defined as the relative ratio of (*ΔR*/*R*_0_) to tensile strain (*ε*), which is calculated by Eq. (3):

3 $${\text{GF}} = \frac{\Delta R/R0}{\varepsilon }$$

As shown in Fig. 3a, it can be clearly observed that with the increase in PDMS coating concentration, the resistance change value of the hybrid membrane decreases significantly at a typical tensile strain, which indicates that the sensitivity of the PCPM-based strain sensor can be significantly affected by the concentration of the PDMS coating. This is due to the contact resistance of the resulted PCPM can experience a gradual increase process with the increase in spray-coated PDMS thickness, in which the PDMS can penetrate into the gap between CNTs to remarkably increase the contact resistance for an increased intrinsic resistance (Fig. S11). Although the GF value of PCPM with 2 wt% PDMS is lower than that of PCPM with 1 wt% PDMS, the electrical stability of PCPM with 2 wt% PDMS is more stable (Fig. S12). The PCPM-based strain sensor (2 wt% PDMS coating) presents a monotonic resistance variation curve with two obvious stages, which is shown in Fig. 3b. The GFs are calculated to be 17.54 (*ε*: 0–30%) and 32.59 (*ε*: 30–60%) coupled with a highly linear response of 0.994 and 0.993, respectively. The response of the strain sensor based on PCPM under different frequencies was also investigated (Fig. 3c). Note that the amplitudes of the output electrical signals remain stable without an obvious change at typical frequencies from 0.01 to 1 Hz, demonstrating a good cycling stability under various frequencies.

Moreover, the real-time cyclic responsiveness of the hybrid film under different strain (*ε* = 1, 5, 10, 20, 30, 40%) was also measured (Fig. 3d). It can be clearly observed that the PCPM-based strain sensor can response sensitively to tiny (1–5%) and large (5–40%) strain and the relative resistance of the five cycles under each strain is stable. In addition to the mechanical sensing performance, when cyclic strain (40% strain) was applied to the sensor, the surface wettability of the sensor can also maintain a stable superhydrophobic characteristics with the WCA value of above 155° under even 1000 times cycles (Fig. 3e). In order to further measure the long-term mechanical sensing stability, the real-time normalized resistance with 5000 times cycles (40% strain and 0.2 Hz) is displayed in Fig. 3f. The result illustrated that the PCPM-based strain sensor could experience a long-time strain and further maintain good electrical stability for further mechanical sensing applications.

Owing to the favourable water-repellent property of PCPM-based strain sensor, it can maintain stable mechanical sensing both in air and water environment (Fig. S13). As shown in Fig. S13a, b, the superhydrophobic sensor can sensitively and steadily detect the finger bending behaviours with different bending angles (15, 30, and 60°). Although the water pressure can weaken the sensitivity of the mechanical sensing, there is still a good ratio of signal to noise under water condition. Besides, when the sensor was exposed to a dynamic environment of intermittent water droplets, the behaviour of finger bending (bending angel of 30°) can be also precisely captured via electrical change. Note that the normalized resistance has a slight increase, which may result from the gravity of the water droplets increasing the original deformation of the sensor (Fig. S13c). For the imperceptible mechanical strain, such as pulse of human, the sensor can effectively detect the pulse signal of the radial artery in air and underwater after 20 push-ups (Fig. S13d–g). As a result, a pulse cycle of about 0.8 s can be calculated according to the arterial pulse waveform, and the corresponding heart rate is 75 beats per minute, which belongs to the normal heartbeat range of healthy people. It can also be observed that the signal intensity increased underwater and the characteristic peaks including those attributed to percussion (*P*), tidal (*T*), and diastolic (*D*) waves of arterial pulses (Fig. S13e–g). Since arterial pulse and heart rate are important health-related indicators, PCPM-based strain sensor can be used as a promising wearable diagnostic device to provide detailed clinical information for a long-term and real-time monitoring of these parameters exposed to water environment.

### Droplet Detection Performance and Direction Balance Sensor

**Fig. 4:**
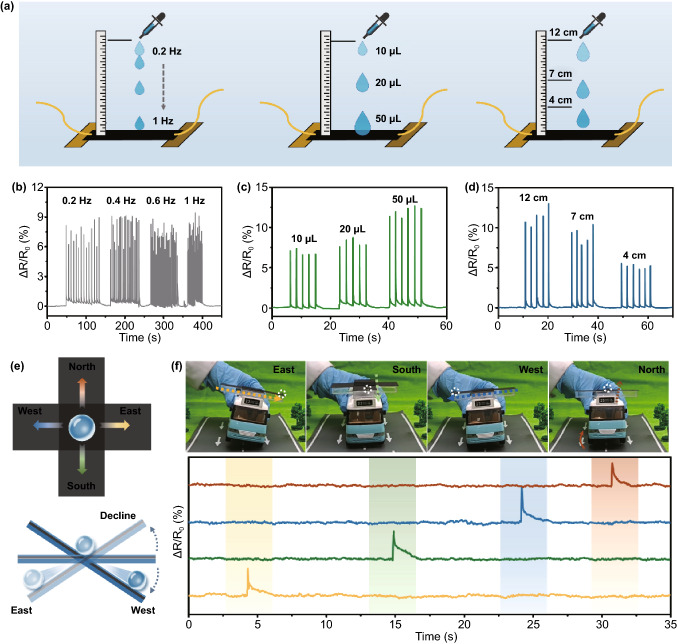
Droplet detection and direction balance detector application. **a** Schematic diagram of droplets at different frequency (0.2, 0.4, 0.6, 1 Hz), different volume (10, 20, 50 μL) and different falling height (12, 7, 4 cm) falling on PCPM. **b**–**d** Relative resistance variation of PCPM-based strain sensor with droplet at different frequency, different volume and different falling height. **e** Schematic diagram of PCPM-based droplet balance detection device. **f** Pictures and corresponding relative resistance variation curves of droplet detector in four directions (east, south, west, north) for unbalance detection, white circles to highlight the water droplets

More interestingly, when PCPM was employed as a self-supported form, it could demonstrate some interesting applications. As shown in Fig. 4a, water droplets with varied dropping frequencies, volumes and dropping heights can be effectively detected. The detection device was fabricated by fixing the two ends of the PCPM on glass substrate with a thickness of 3 mm with double-sided tape. The size of the PCPM-based sensor is 5 × 20 mm^2^, and the electrode was encapsulated by PDMS. Figure 4b–d shows the corresponding normalized resistance curves of the PCPM-based sensor versus varied frequencies, volumes and heights.

Based on this advantage, a superhydrophobic self-supported PCPM-based droplet balancing sensor was prepared. Balance detection is crucial in daily life. For example, tremendous autotruck need to be tested for balance as it leaves the factory to prevent dangerous accidents. As shown in Figs. 4e and S14, the balancing sensor consists of two parts: self-supporting substrate (bottom) and superhydrophobic PCPM film (top). The self-supported substrate is a hollow acrylic with a crisscross shape, which is composed of two rectangles with a size of 30 × 90 mm^2^. We coated the edges of the cross substrate with PDMS to fix the self-supporting film. In the middle is a 30 × 30 mm^2^ candle ash superhydrophobic glass plate, which is the initial moving area of the droplet. The four directions of east, west, south and north correspond to four self-supported PCPM superhydrophobic sensors with a size of 30 × 30 mm^2^. When a droplet rolls over a certain direction, the signal output in that direction. When the droplet balancing sensor is unbalanced in the four directions of east, west, south and north regularly, the corresponding signals can be successively detected (Fig. 4f and Movie S1).

### Multifunctional Smart Umbrella for Weather Monitoring

The interfacial fabrication method of PDMS film enabled controllable thickness of the film, allowing thin and adaptive characteristics of resulted PCPM. It can be easily transferred onto structural surface and spread smoothly to form a self-supported and conformal structure. As a proof of concept, a miniature umbrella composed of self-supported PCPM was designed in our system. Owing to the favourable superhydrophobicity, thin, conductive and elastic properties, and the integrated smart umbrella can realize high resolution of water droplets and correlated frequencies/intensity.

**Fig. 5:**
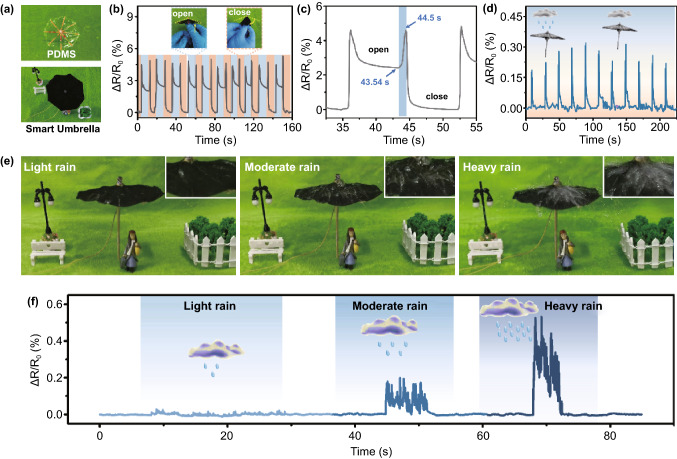
Self-supporting smart umbrella detects rainfall. **a** Pictures of self-supporting smart umbrella. **b**–**c** Relative resistance value of the smart umbrella in the opening and closing process and time response of intelligent umbrella between opening and closing. **d** *ΔR*/*R*_0_ versus time curve of intelligent umbrella under rainy or cloudy days. **e**–**f** Photos of self-supporting intelligent umbrella for monitoring rainfall and the corresponding relative resistance value of the smart umbrella in light rain, moderate rain and heavy rain

Therefore, we prepared a smart umbrella by imitating the structure of the umbrella (Figs. 5a and S15). To explore the dynamic response performance of the smart umbrella, the normalized resistance in the opening and closing process of the smart umbrella is measured in Fig. 5b. To further investigate the robustness of PCPM-based smart umbrella, a larger smart umbrella was constructed to conduct 200 times of open and close cycles (Fig. S16). It is clearly observed that the smart umbrella experiences a good cyclic stability of electrical signal during the opening and closing process, demonstrating a favourable mechanical endurance of the PCPM. Furthermore, we compared the mechanical properties of PCPM films with and without PDMS coating. The result shows that the PCPM film coated with PDMS exhibits favourable mechanical properties, with a tensile strength of 1.5 MPa (Fig. S17). When the smart umbrella is opened, the PCPM is stretched to a certain extent, and subsequently, the contact area among CNTs decreases, resulting in a remarkable increase of resistance. On the contrary, the resistance response decreases when the smart umbrella is closed. Due to the favourable sensing performance of PCPM-based strain sensor, the signal change in the opening and closing process of the smart umbrella only takes 0.96 s (Fig. 5c), which enables the smart umbrella to open and close timely according to the needs. Figure 5d shows the cyclic normalized resistance variation curves of the smart umbrella to detect the weather condition (cloudy or raining). Note that the superhydrophobic feature has enabled an effective rejection of dropped water droplets for precise detection of water dropping information. When a simulated raining was applied on the smart umbrella, the smart umbrella presented a strong signal response, and the signal was back to the baseline sharply when the rain stopped.

The good sensitivity of the smart umbrella to water droplets allows it to detect the real-time weather condition. The pictures of the smart umbrella for detecting rainfall are shown in Fig. 5e. In the experiment, the sprinkler was used to control rainfall (Movie S2). It can be clearly observed from Fig. 5f that the smart umbrella can not only detect for a long time, but also clearly distinguish rain intensity, including light, moderate rain or heavy states. Based on the above experimental results, we can programme the smart umbrella in the future. When the first drop of rain is detected, the smart umbrella will open automatically and close automatically when the rain stops. As is known, rainy weather is usually accompanied by wind; thus, the influence of wind on PCPM-based smart umbrellas is also explored (Fig. S18). In the experiment, we used the fan to simulate the wind. It can be observed in Fig. S18, the current change of wind (*I* =  ~ 0.006 mA) has less influence on PCPM-based smart umbrella compared with rain (*I* =  ~ 0.07 mA). Moreover, the smart umbrella can monitor the amount and time of rainfall in real time, which provides great convenience for our life.

**Fig. 6:**
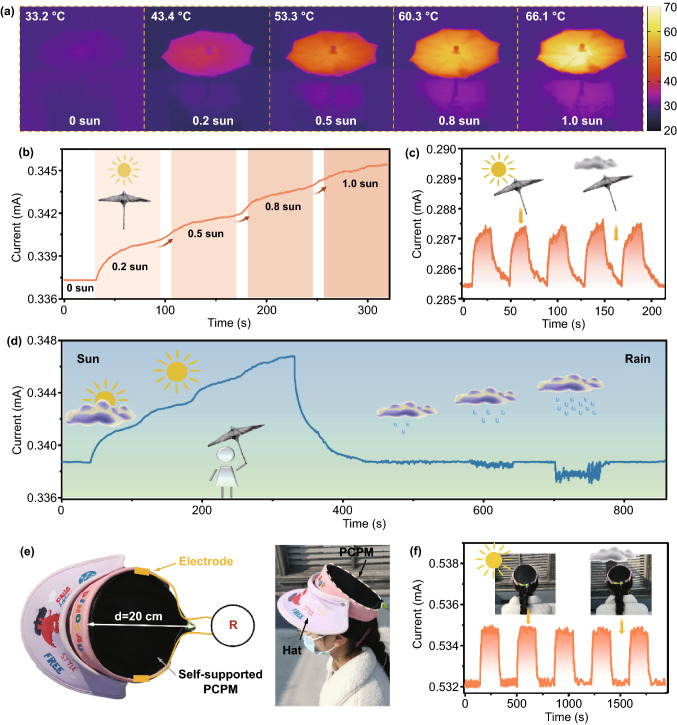
Self-supporting smart umbrella detects light intensity. **a** IR images of intelligent umbrella at varied light intensity. **b** Current–time curve of intelligent sun umbrella at varied solar intensity. **c** Current–time curve of the smart umbrella in the presence or absence of sun light (0.5 sun). **d** Current–time curve of the smart umbrella for identifying the weather in a day. **e** Pictures of the structure of self-supporting smart sunbonnet. **f** Current–time curve in the presence or absence of sun light (0.5 sun)

In our system, owing to the introduction of CNTs film with favourable photothermal property, the integrated umbrella was endowed with good solar-to-thermal conversion. As displayed in Fig. 6a, the equilibrium temperature represented a positive correlation with the applied solar intensity. The maximum temperature can reach up to 66.1 °C under 1 sun. Note that in the absence of other conditions, the temperature has a quantitative relationship with the resistance of CNTs, as shown in Eq. (4):

4 $$R = - {\text{Ln}} \,T (T > 1 K)$$

The surface temperature of the smart umbrella remains a stable value without sunlight, and the free *π* electrons in CNTs move along the molecular chain under the applied voltage, leading to unchanged current signal. When solar was applied, the temperature of the smart umbrella gradually increased and the electrons in the outer layer of carbon atoms gained enough energy and are no longer restricted in a certain region. Meanwhile, the thermal movement of molecules accelerates, making the band gap width in CNTs smaller. As a result, more electrons in the valence band gain energy to transition to the conduction band, increasing the number of *π* electrons and thus increasing the current (Fig. S19). Therefore, the semiconductive CNTs can be effectively boosted by the sun illumination, resulting in an efficient improvement of the conductivity of PCPM. With the increase in solar intensity ranging from 0.2 to 1 sun, the balanced normalized current can experience a prominent increase from 0.337 to 0.345 mA (Fig. 6b). In addition, we also conducted the cyclic illumination experiments on the smart umbrella, presenting a good response to applied solar illumination (Fig. 6c).

The capability of solar response can inspire us to exploit the functions of umbrella to effectively monitor the weather (sunny day and its solar intensity, rainy day and its raining intensity). Based on this, changeable weather in certain day was simulated, including the sunny state with gradually increased solar intensity, cloudy state and the rainy state from light to heavy rain. As displayed in Fig. 6d, the smart umbrella can experience a corresponding current change, enabling a gradual solar-enhanced current elevation, a quick current decrease without the solar illumination and quick/sensitive response to the rain with different intensity. The weather-response smart umbrella is expected to demonstrate significant potentials in dynamic detection of changeable weather. Furthermore, benefitted from the interface-enabled fabrication method, the PCPM can be editable into various shapes (Fig. S20), such as stars, fish, and numbers. Furthermore, a large-area PCPM could be constructed in a facile and controllable way. As shown in Fig. S21, a PCPM film with a diameter of ~ 30 cm was prepared and could be further transferred to substrate as a self-supported one, demonstrating the capability of large-area fabrication. Similar to the umbrella, a demo of wearable smart sunhat with the diameter of 20 cm was developed in our experiment (Fig. 6e). In outdoor experiment, the sunhat was placed in the sun and in the shade for 3 min, respectively, and its current–time curve is shown in Fig. 6f. The result illustrated that the solar illumination could be effectively captured. And there was stable and repeatable current response to the solar, demonstrating potential applications in the wearable smart devices.

### Multifunctional Smart Umbrella for Water Rescue and Sensing

**Fig. 7:**
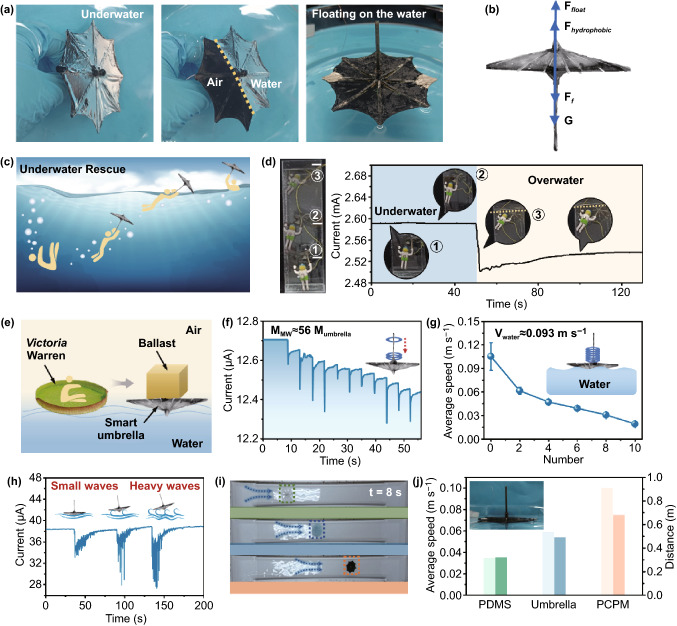
Underwater rescue and sensing application. **a** Photograph of the smart umbrella underwater, in the air and floating on the water. **b** Schematic of the force of the smart umbrella underwater. **c** Schematic of the whole process of underwater rescue with smart umbrella. **d** Diagram of intelligent underwater rescue model and current–time curve in the process of rising. **e** Diagram and inspiration of the Victoria Warren carrying people. **f** Current–time curve of smart umbrella on small, medium and large waves water surfaces. **g** The average velocity of smart umbrella carrying different number of iron rings on the water. **h** Current–time curve of smart umbrella on small, medium and large wave water surfaces. **i** The position of pure PDMS umbrella, pure oil paper umbrella and PCPM umbrella at *t* = 8 s when the flow velocity is 0.093 m s^−1^ (the total distance is 1 m, starting at the same time). **j** The average speed and distance of PDMS umbrella, pure oil paper umbrella and PCPM umbrella when the flow velocity is 0.093 m s^−1^

Since the PCPM with superhydrophobic feature can effectively repel water, it can further function as a flexible tool for water rescue. The strong repulsive force can dominantly drive the underwater smart umbrella to rapidly float on water surface. As shown in Fig. 7a, the smart umbrella shows a mirror-like phenomenon beneath water, which indirectly reflects its good superhydrophobic feature. When the smart umbrella was put into water, it could move onto surface quickly and float steadily on the water. In order to further explore the rapid floating mechanism of the smart umbrella, we carefully analysed the generated forces on umbrella. As depicted in Fig. 7b, the smart umbrella is subjected to four forces, namely the gravity of the umbrella model (*G*), resistance (*F*_f_), hydrophobicity (*F*_hydrophobic_) and upward buoyancy (*F*_float_). There is a certain relationship between the motion acceleration (*a*) of the smart umbrella and the forces as shown in Eq. (5):

5 $$a = \left( {F_{{{\text{float}}}} + F_{{{\text{hydrophobic}}}} - G - F_{{\text{f}}} } \right) / M_{{{\text{model}}}}$$

where *F*_hydrophobic_ is determined by the amount of air stored on the surface of the smart umbrella, which is a fixed value in the water. *M*_model_ is the mass of the loaded object, and *G* is gravity, both of which are fixed. *F*_f_ is also a constant value because it is related to the stressed area. Therefore, the acceleration of the smart umbrella is constant as it rises through the water. The schematic of underwater rescue is shown in Fig. 7c. The smart umbrella and the toy model was fixed and forced into the simulated underwater environment at a depth of 40 cm. Prior to the water rescue experiment, magnets were loaded on the back of the toy model to maintain a suspended state in the water (The density is analogous to human density (1 ~ 1.1 g cm^−3^)) (as shown in Fig. 7d inset). Therefore, very little force can effectively break the balance. When the toy model is connected to the umbrella, it could experience a fast floating process with the aid of umbrella. Meanwhile, the whole process was also effectively monitored via the change of normalized resistance of the umbrella (Fig. 7d and Movie S3).

Moreover, similar to the Victoria Warren, the floated umbrella could load objects with many times weight than itself (Fig. 7e). Therefore, the capability of loading is also explored in Fig. 7f. The total weight of the iron ring that successively put on the floating umbrella surface is about 19 g and the umbrella skeleton and PCPM membrane weigh about 1.5 and 0.366 g, respectively. A staircase-like current signal could be clearly observed, demonstrating a real-time precise detection of the load. The calculated maximum load could reach up to more than 56 times than its own weight. More interestingly, the average moving speed of the smart umbrella could be effectively adjusted by controlling the loaded ring quantities (number = 0, 2, 4, 6, 8, 10) (Fig. 7g).

Owing to the thin and elastic feature of PCPM, the floated umbrella can also detect the waves intensity. Through the information of current signals, the small or heavy waves could be successfully identified (Fig. 7h). On the other hand, the superhydrophobic feature can also accelerate the moving speed of the miniature umbrella (Fig. 7i, j). Compared with the commercially available toy umbrella and PDMS umbrella, the superhydrophobic one shows a fast moving speed at the same simulated water flow velocity, providing a potential alternative for water rescue.

## Conclusion

In summary, we have designed a self-supported film featured with elastic, thin, conductive and superhydrophobic characteristics. The integration of mechanical, electrical and wetting properties into one system can endow the achieved film with exceptional features. With the programmable adjustment of surface wettability and corresponding sensory capability, the self-supported film integrated smart umbrella can dynamically sense the simulated raining scene with changeable frequencies and intensity. When exposed to sunlight, it can effectively detect the change of solar intensity via current signal. In addition to the monitoring of weather, the smart umbrella can further realize a multifunctional water rescue tool for fast underwater floating, load weight, rapid transportation and real-time current monitoring of the whole process. As a result, the self-supported film enabled umbrella system is expected to be an alternative for unconventional water-related applications, demonstrating significant potentials in the development of multifunctional sensory system, soft actuators under water condition.

## Supplementary Information

Below is the link to the electronic supplementary material.

Supplementary file1 (MP4 10102 kb)

Supplementary file2 (MP4 10654 kb)

Supplementary file3 (MP4 10809 kb)

Supplementary file4 (PDF 1853 kb)
