# Supplementary material for: Bioinspired Nanostructured Superwetting Thin-Films in a Self-supported form Enabled “Miniature Umbrella” for Weather Monitoring and Water Rescue
Source: Nanomicro Lett. 2021 Dec 13;14:32. doi: 10.1007/s40820-021-00775-4 (PMC8669048; doi:10.1007/s40820-021-00775-4)
Supplement: Supplementary file 4 — Supplementary file4 (PDF 1853 kb) [file 40820_2021_775_MOESM4_ESM.pdf]

Supporting Information for

Bioinspired Nanostructured Superwetting Thin-Films in a Self-supported form Enabled "Miniature Umbrella" for Weather Monitoring and Water Rescue

Shan Li[1, 2], Peng Xiao[1, 2, *], Wei Zhou[1, 2], Yun Liang[1, 2], Shiao-Wei Kuo[3], Tao Chen[1, 2, *]

[1]Key Laboratory of Marine Materials and Related Technologies, Zhejiang Key Laboratory of Marine Materials and Protective Technologies, Ningbo Institute of Materials Technology and Engineering, Chinese Academy of Sciences, Ningbo 315201, P. R. China

[2]School of Chemical Sciences, University of Chinese Academy of Sciences, 19A Yuquan Road, Beijing 100049, P. R. China

[3]Department of Material and Optoelectronic Science, Center of Crystal Research, National Sun Yat-Sen University, Kaohsiung 804, Taiwan, P. R. China

*Corresponding authors. E-mail: xiaopeng@nimte.ac.cn (Peng Xiao); tao.chen@nimte.ac.cn (Tao Chen)

Supplementary Figures

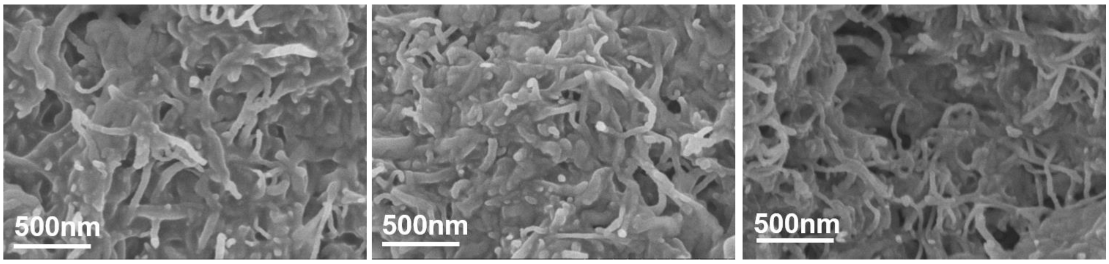

Fig. S1 SEM images of the hybrid film (without coating PDMS) with one layer, two layers, three layers CNTs transferred

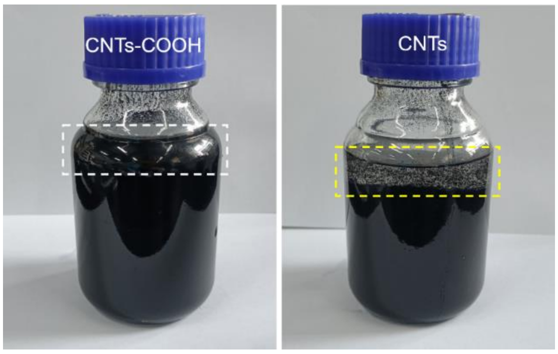

Fig. S2 Photo of CNTs with -COOH and unmodified CNTs dispersed in ethanol solution

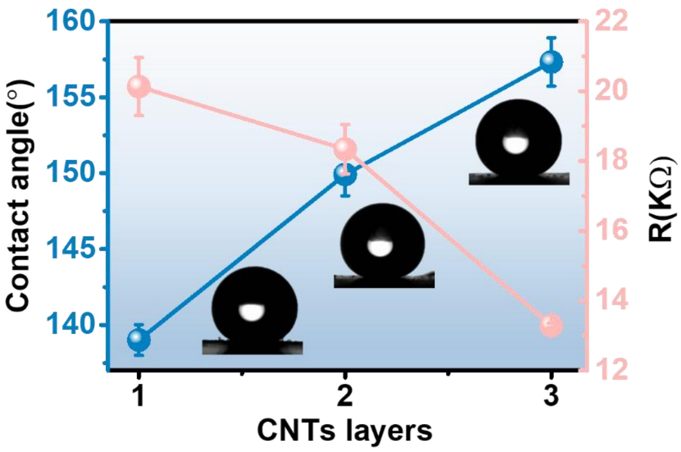

Fig. S3 Water contact angle and relative resistance variation of the hybrid film (without coating PDMS) with different CNTs layers (one, two and three)

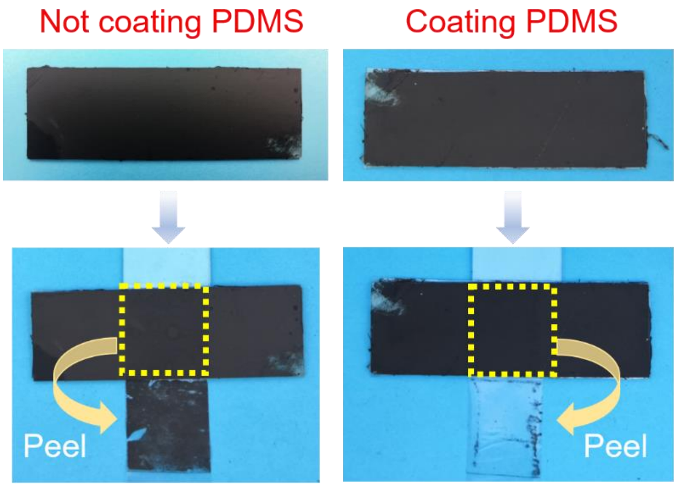

Fig. S4 Schematic diagram of commercial tape peeling after adhesion of film surface without PDMS coating and with PDMS coating

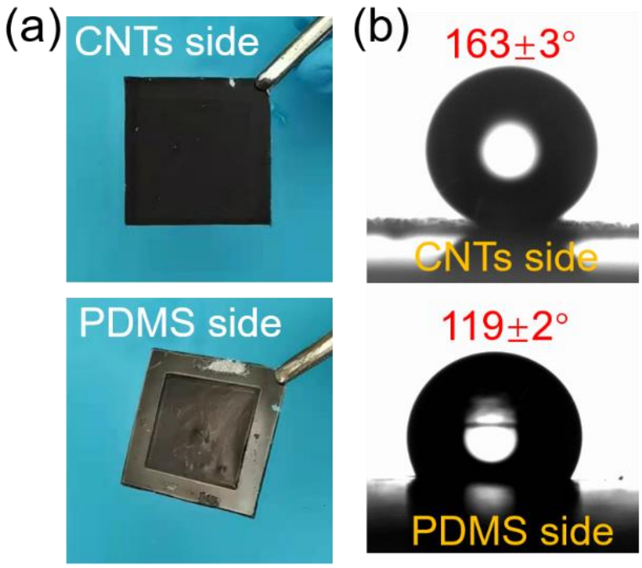

Fig. S5 Photos **a** and Water contact angles **b** of the CNTS side and PDMS side

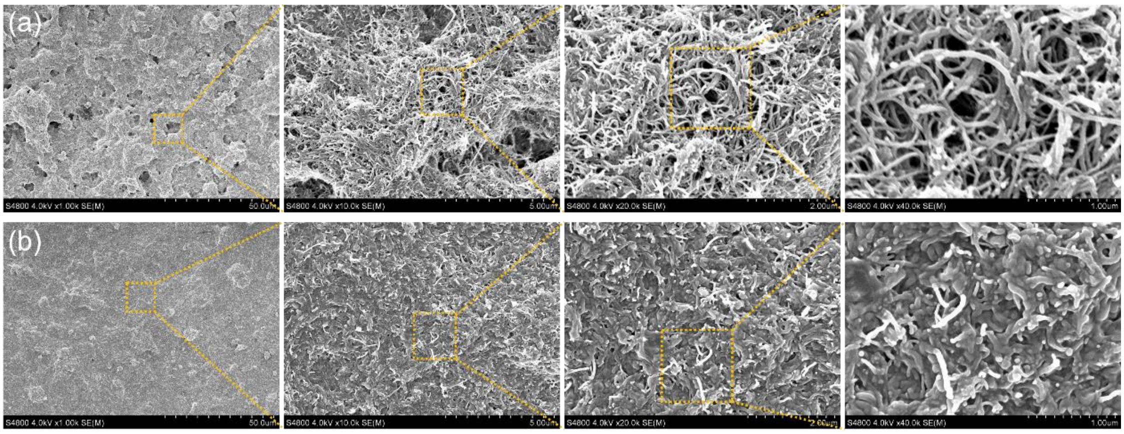

Fig. S6 a SEM images of CNTs/PDMS at different rates without PDMS coated. **b** SEM images of PCPM sprayed with 2 wt% PDMS at different rates

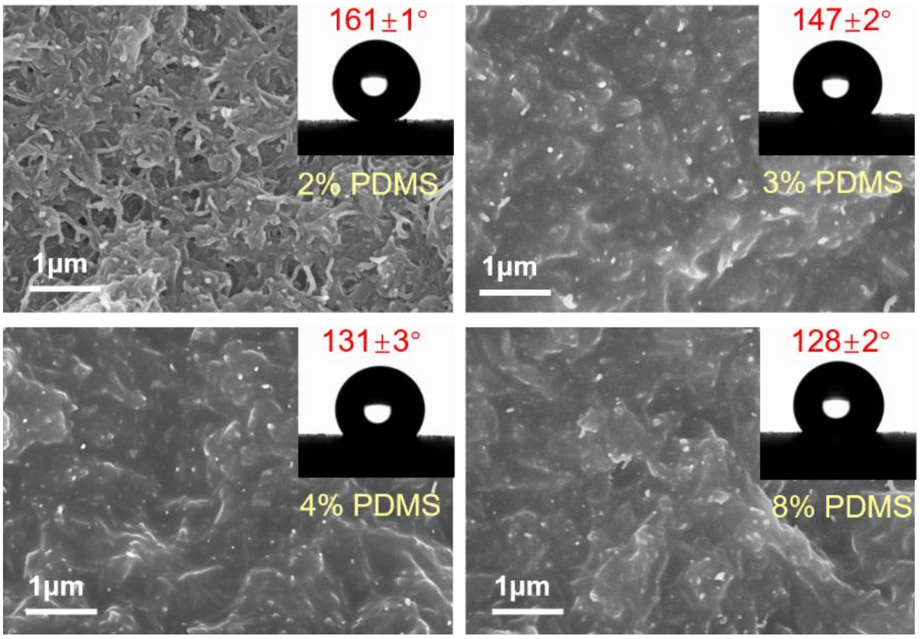

Fig. S7 SEM and water contact angle images of the PCPM coated with different mass concentrations PDMS (wt = 2%, 3%, 4%, 8%)

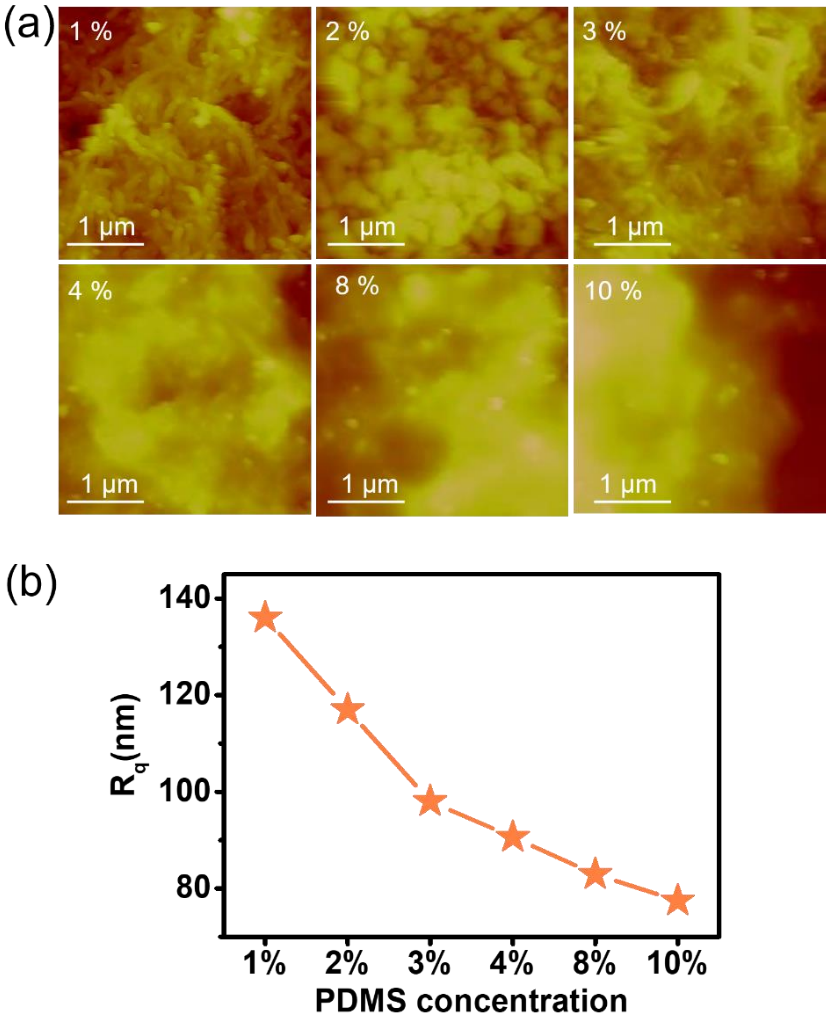

Fig. S8 a AFM image of the different concentration PDMS. **b** Roughness curves of different concentrations of PDMS

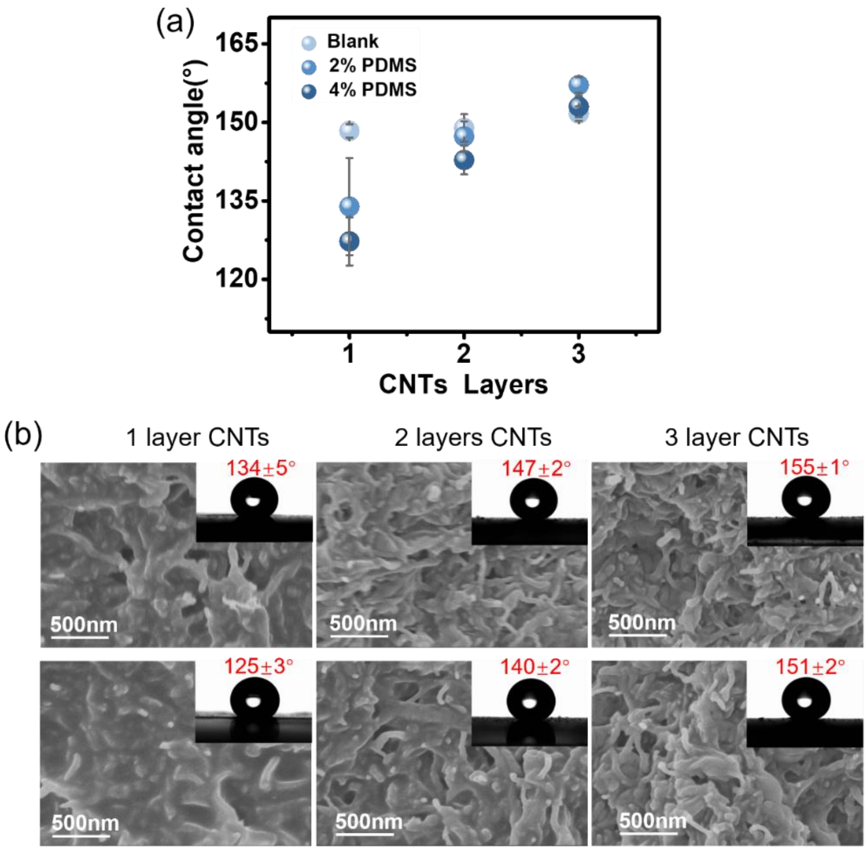

Fig. S9 **a** Water contact angles of the PCPM with different CNTs layers were coated with 2 wt% and 4 wt % PDMS, respectively. **b** SEM images of PCPM hybrid film with different CNTs layers

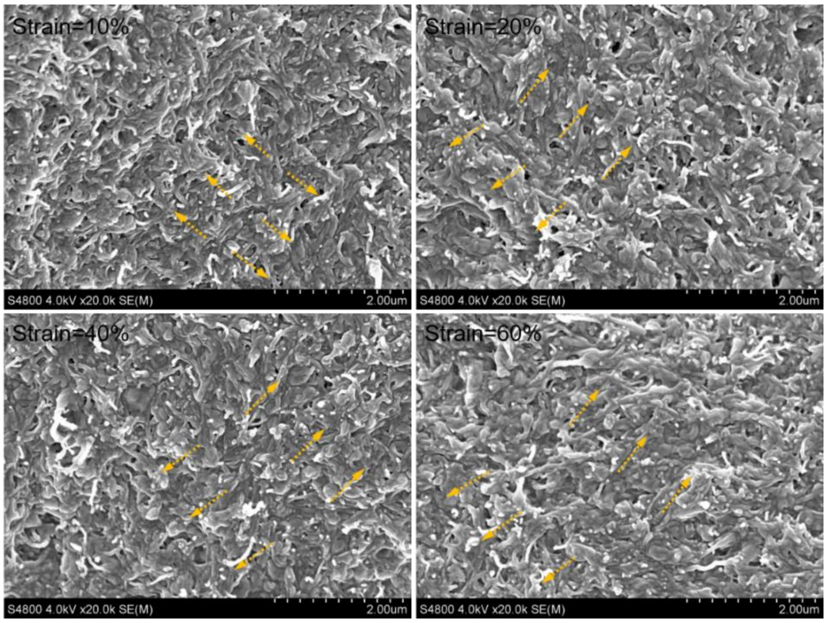

Fig. S10 SEM images of PCPM films (2%) under tensile strain of 10%, 20%, 40% and 60%

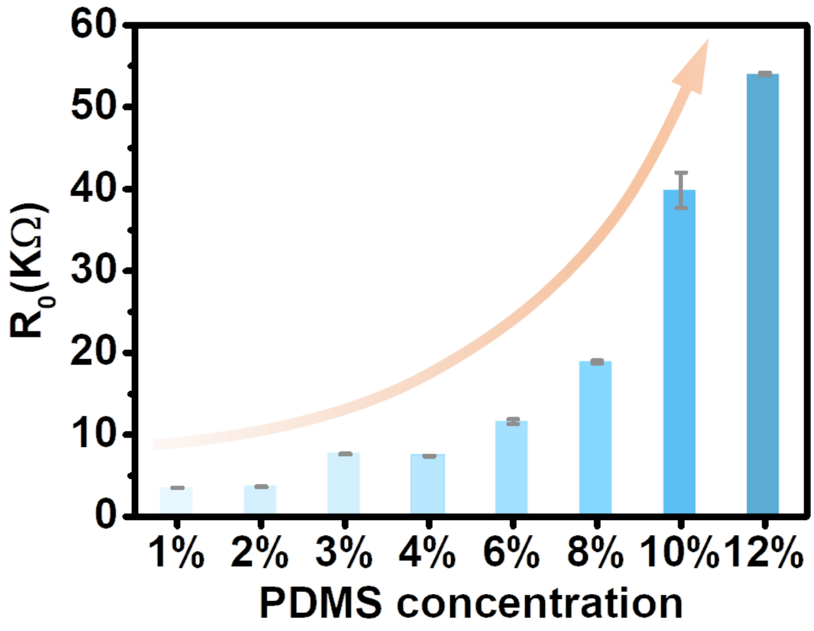

Fig. S11 The change of initial resistance of PCPM coated with different concentrations of PDMS

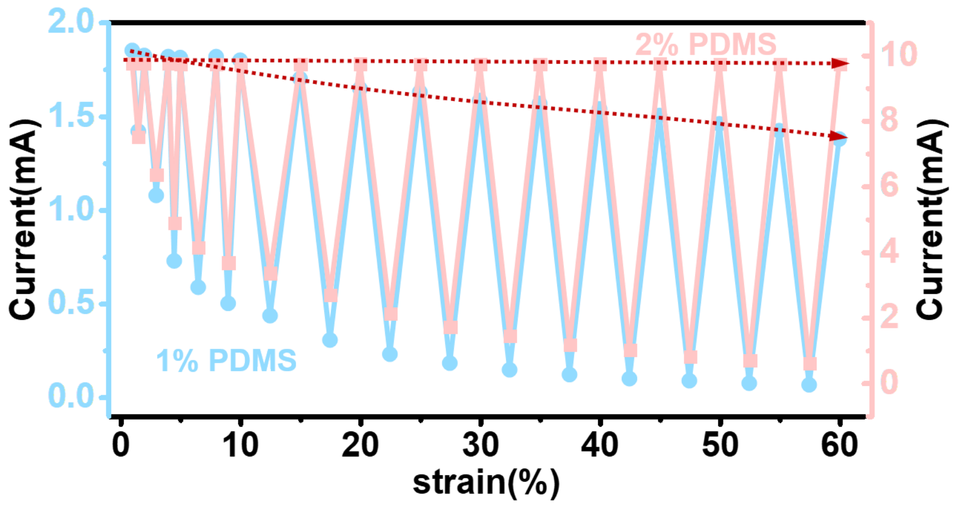

Fig. S12 The current of hybrid films coated with 1 wt% and 2 wt% (mass concentrations) PDMS under different deformations returned to the initial state (ε = 40%)

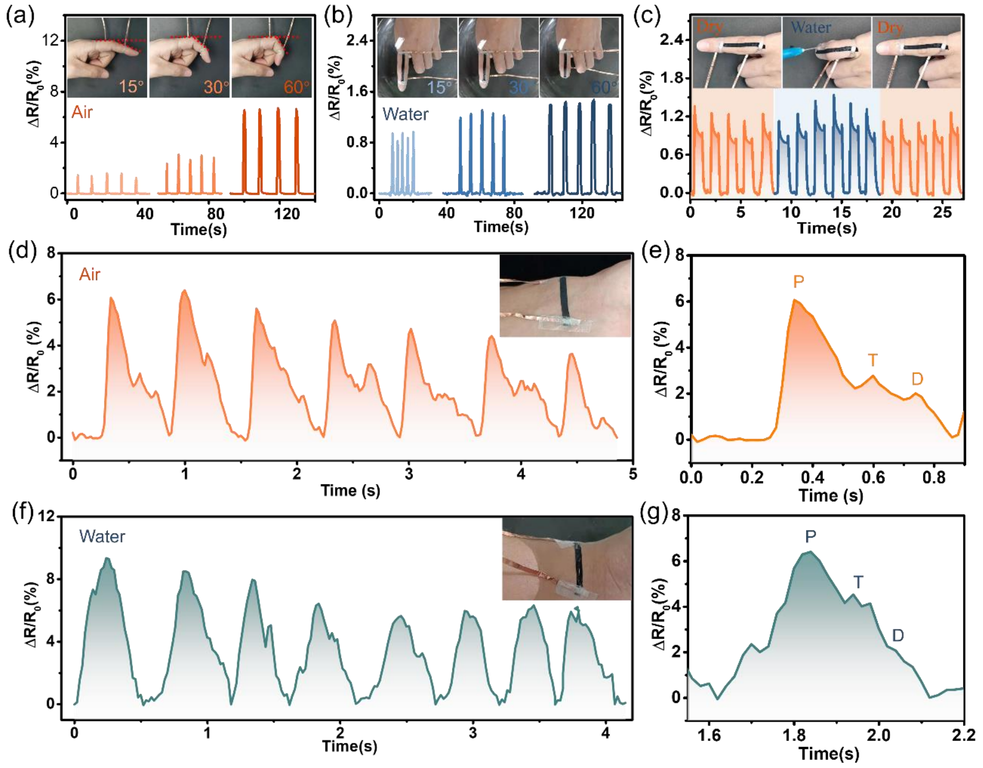

Fig. S13 Detection of human movement in air and water. Relative resistance variation of PCPM strain sensor during finger bending and releasing behavior in air **a** and water **b**. **c** $\Delta R/R_0$ versus time curve of PCPM strain sensor under a constant drip condition. Relative resistance variation of PCPM strain sensor during strain detection of human motion respectively in air **d** and water **f** and close up of the corresponding region in **e** and **g**

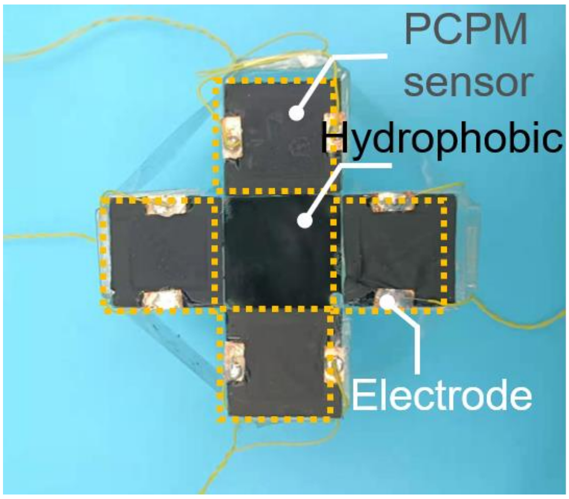

Fig. S14 Diagram of composition of droplet balance sensor. It is composed of five parts, the middle is a 3×3 cm^2 glass plate on the candle flame burning 2 min to get the super-hydrophobic structure, the purpose is to make the liquid droplets can roll smoothly in four directions. The four PCPM sensors in the east, west, south and north are respectively welded with copper foil and tin wires at the left and right ends. During detection, the four directions are connected with the electrochemical workstation.

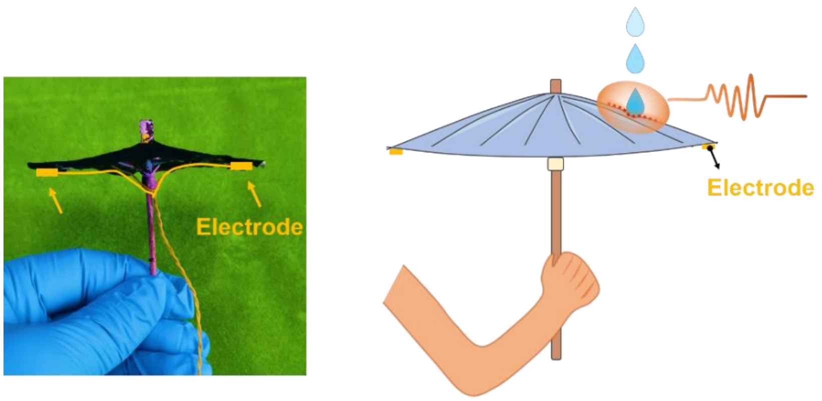

Fig. S15 PCPM smart umbrella diagram. Electrodes are connected to insides of the umbrella and sealed with PDMS (left). Noted that a hole should be cut at the top of the umbrella to make the surface of the PCPM umbrella smooth. Working mechanism diagram of PCPM smart umbrella. When raindrops fall on the surface of the smart umbrella, the conductive film deforms, causing the current to change.

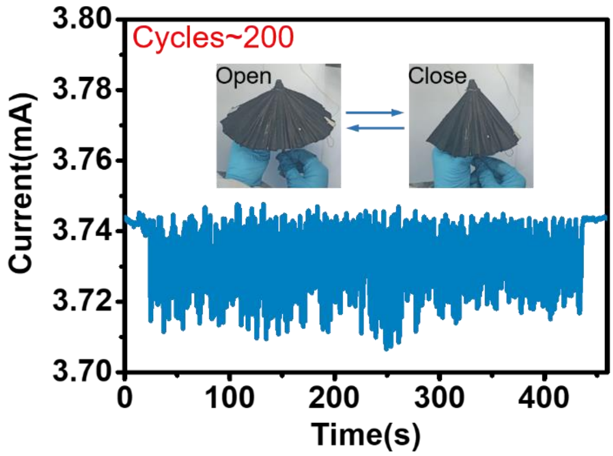

Fig. S16 Mechanical performance diagram of the PCPM smart umbrella (diameter≈17 cm) opening and closing about 200 cycles.

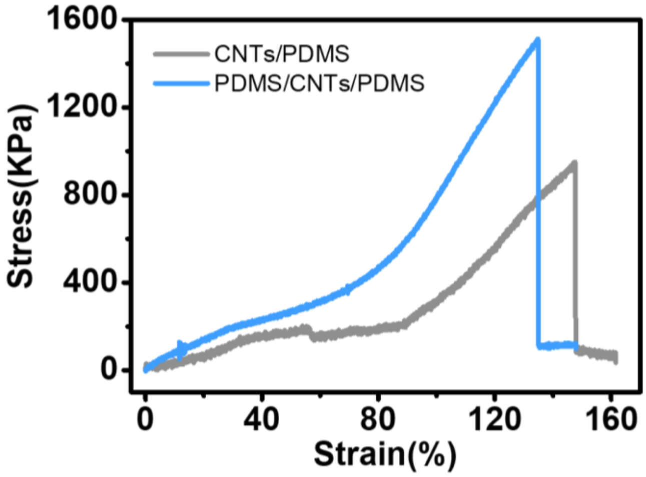

Fig. S17 Mechanical properties of films coated with PDMS (2%) and without PDMS

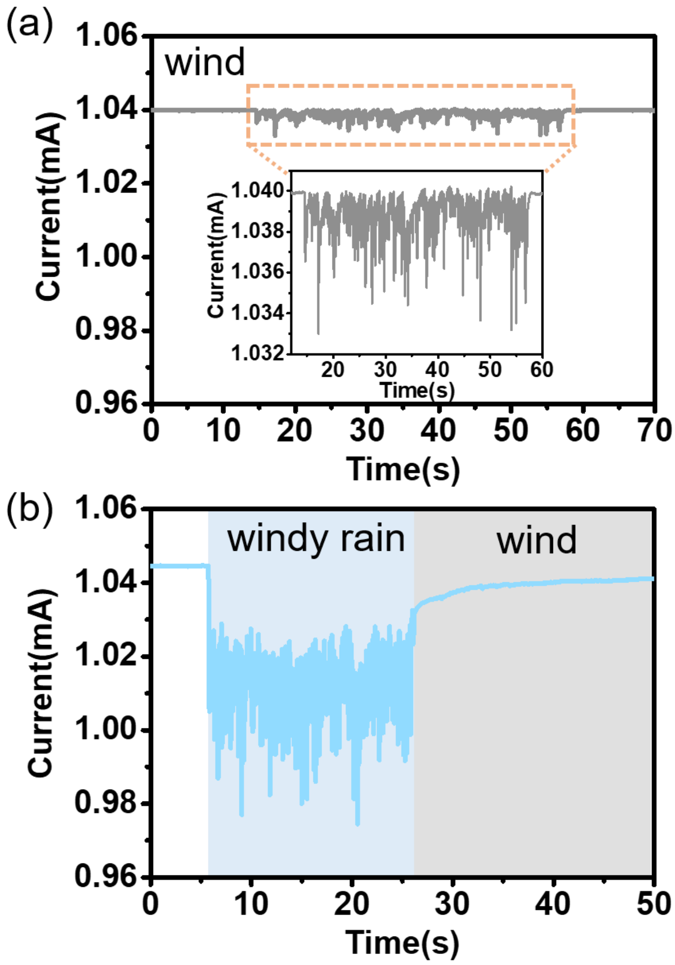

Fig. S18 a Current-time curves of PCPM smart umbrella on windy day. **b** Current-time curve of PCPM smart umbrella windy rain day

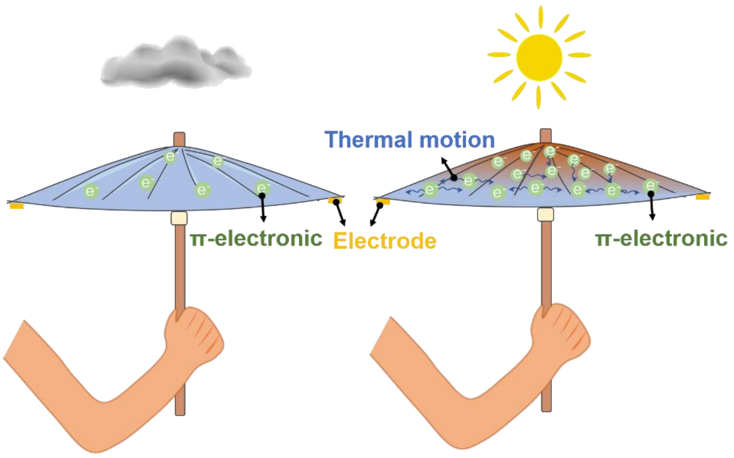

Fig. S19 Working mechanism diagram of the PCPM smart umbrella. When the smart sun umbrella is not illuminated, the free π electrons in CNTs move along the molecular chain under the applied voltage. The number of electrons is stable and the current unchanged. When the smart sun umbrella is illuminated, under the function of photothermal effect, the thermal motion of electrons intensifies, the number of π electrons increases, and the current increases.

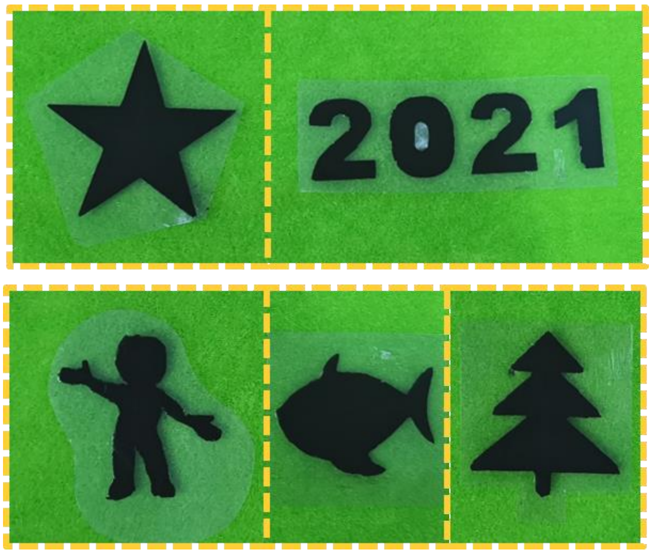

Fig. S20 Patterning of PCPM. PCPM was transferred to PET and then cut ted to get various patterns, including stars, numbers, small person, fish and trees.

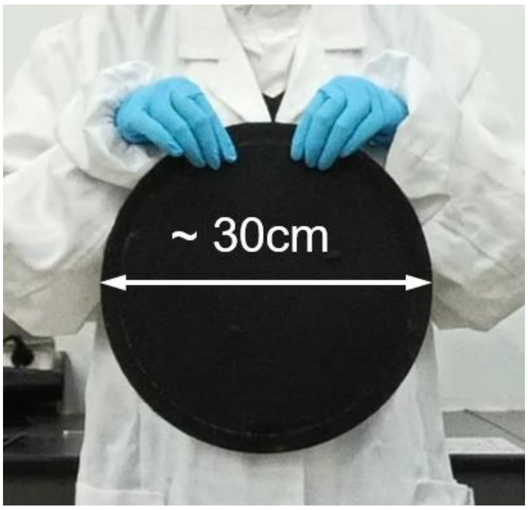

Fig. S21 Photograph of PCPM film with diameter of about 30 cm
